# Recovery Estimation of Dried Foodborne Pathogens Is Directly Related to Rehydration Kinetics

**DOI:** 10.1371/journal.pone.0160844

**Published:** 2016-08-05

**Authors:** Emilie Lang, Fiona Zoz, Cyril Iaconelli, Stéphane Guyot, Pablo Alvarez-Martin, Laurent Beney, Jean-Marie Perrier-Cornet, Patrick Gervais

**Affiliations:** 1 Unité Mixte de Recherche—Procédés Alimentaires et Microbiologiques (UMR PAM), Université de Bourgogne Franche-Comté, AgroSup Dijon, 1, Esplanade Erasme, 21000, Dijon, France; 2 Novolyze, 50 rue de Dijon, 21121, Daix, France; University of Campinas, BRAZIL

## Abstract

Drying is a common process which is used to preserve food products and technological microorganisms, but which is deleterious for the cells. The aim of this study is to differentiate the effects of drying alone from the effects of the successive and necessary rehydration. Rehydration of dried bacteria is a critical step already studied in starter culture but not for different kinetics and not for pathogens. In the present study, the influence of rehydration kinetics was investigated for three foodborne pathogens involved in neonatal diseases caused by the consumption of rehydrated milk powder: *Salmonella enterica* subsp. *enterica* serovar Typhimurium, *Salmonella enterica* subsp. *enterica* serovar Senftenberg and *Cronobacter sakazakii*. Bacteria were dried in controlled relative humidity atmospheres and then rehydrated using different methods. Our results showed that the survival of the three pathogens was strongly related to rehydration kinetics. Consequently, rehydration is an important step to consider during food safety assessment or during studies of dried foodborne pathogens. Also, it has to be considered with more attention in consumers’ homes during the preparation of food, like powdered infant formula, to avoid pathogens recovery.

## Introduction

Drying is an environmental and technological perturbation involving a water transfer from a liquid state to a gaseous state, following the a_W_ gradient which allows the transformation from a liquid to a solid and dried product. Water activity or a_W_ represents the available water for chemical and biochemical reactions and is theoretically comprised between 0 (no water) and 1 (pure water). During drying, the water activity of products is reduced, preventing bacterial growth and below an a_W_ of 0.6, no microorganisms can grow. This is the reason why drying is a common method used for the preservation of food products or microorganisms of technological interest such as lactic acid and probiotic bacteria. Several methods can be used to reduce a_W_ and, consequently, to dry food or microorganisms. Common drying processes are spray-drying, fluidized bed drying and freeze-drying [1,2] and are used to conserve dried food products such as herbs, spices or milk. Moreover, drying has a deleterious effect on cells. Indeed, at the beginning of the drying process, evaporation of water causes an increase in the osmotic pressure of the extracellular medium and, consequently, provokes a large cellular water outflow from the cells creating then an osmotic stress which is damaging for cells [3]. Afterward, in the case of drying conducing to products of low a_W_, the water removal from extracellular and intracellular media will provoke a direct exposition of cells to oxygen in the air which generates reactive oxygen species in cells and the accumulation of free radicals, creating oxidative stress [3,4]. Damages caused by this stress affect macromolecules and membranes [3,5–9]. Nevertheless, even if drying involves cellular injuries to bacterial cells, dried foods are non-sterilized foods [10] and a large range of microorganisms can survive drying such as fungi, like *Aspergillus* spores, bacteria, like *Salmonella* spp., and viruses, like Norovirus [10,11]. Some of them can be dangerous for consumers’ health and this is the reason why a great deal of research is focused on the development of specific decontamination processes [12] for the purpose of achieving a minimal microbial load in dried food according to legal requirements and for consumer health protection.

Assessment of microbial survival in dried products involves a rehydration step that is necessary in the method to restore cellular activities, permitting to reach a physiological a_W_. The influence of this step has already been studied for the optimization of preservation processes of lactic acid bacteria and flora with technological purpose in a dried form [1]. For example, Morgan *et al*. (2006) reviewed the impact of rehydration of freeze dried lactic acid bacteria and presented it as the final critical step for the revival of cells after drying. Nevertheless, the rehydration kinetics has not been investigated in foodborne pathogens. Moreover, this step has been neglected in the case of pathogens contaminating dried foods or environments. Accordingly, the current method for microbial safety assessment of dried food products is based on their rehydration in a liquid buffer before diluting and spreading for cultivability measurement. However, the rehydration mode, and particularly rehydration kinetics, could drastically impact the cultivability measurement and could lead to erroneous estimation of pathogenic bacteria. This may pose a risk for the consumer due to an underestimation of pathogenic bacteria. Moreover, dried foods are often rehydrated by the user and therefore the impact of rehydration could also directly impact food contamination and consumer health.

Low-moisture foods are not sterile, therefore they can be involved in foodborne outbreaks. For example, *Enterobacteriaceae*, such as *Escherichia coli* O157:H7, *Salmonella enterica* and also *Cronobacter sakazakii*, are implicated in outbreaks linked low-moisture foods, such as spices, flour, powdered infant formula (PIF) or herbs [10,11,13,14]. Among bacteria found in dried food products, *Salmonella enterica* is one of the most represented foodborne bacteria every year across the world. Regarding *Cronobacter sakazakii*, even if incidence is low, it is involved in neonatal meningitis with a death rate up to 80%, hence the importance of assessing microbial contamination.

In the present study, we aimed to distinguish the effects of drying alone from the effects of the successive and necessary rehydration in order to investigate the effect of rehydration kinetics after drying on the survival of three pathogens: *Salmonella enterica* subsp. *enterica* serovar Typhimurium, *Salmonella enterica* subsp. *enterica* serovar Senftenberg and *Cronobacter sakazakii*.

## Materials and Methods

### Bacterial strains

*Salmonella enterica* subspecies *enterica* serovar Typhimurium DT104 DSM 10506, *Salmonella enterica* subspecies *enterica* serovar Senftenberg DT104 DSM 10062 and *Cronobacter sakazakii* CIP 103183T strains were used for this study. Bacteria were inoculated on Tryptic Soya Agar (TSA, Sigma-Aldrich, Saint-Quentin-Fallavier, France) at 37°C (+/‒ 0.1°C) for 24 h, subsequently five colonies of each bacterium were inoculated in 50 mL of Tryptic Soya Broth (TSB, Sigma-Aldrich, Saint-Quentin-Fallavier, France) and incubated for 8 h at 37°C (+/‒ 0.1°C). Suspensions were then diluted in 50 mL of fresh TSB in order to adjust an Optical Density at 600 nm (OD_600_, measured by using a Thermo Scientific, Spectronic 200, Villebon sur Yvette, France) of 0.01 (corresponding approximately to 10^6^ CFU/mL) before incubation for 14 h at 37°C (+/‒ 0.1°C) to reach stationary growth phase cultures.

### Drying conditions

#### Drying atmosphere

For the desiccation of the cellular suspensions, hermetic plastic boxes (20 × 13 × 6 cm) were used, with saturated salt solutions at the bottom which regulated the a_W_ and therefore the atmosphere RH. Saturated salt solution of lithium chloride, potassium acetate, potassium carbonate and magnesium nitrate (all from Sigma-Aldrich) were used to reach an a_W_ of 0.11, 0.25, 0.44 and 0.58, respectively, checked using an a_W_-meter (+/‒ 0.003) (Aqualab, Dardilly, France). In the hermetic boxes, a_W_ permitted to maintain a RH of 11%, 25%, 44% and 58%, respectively, checked with RH sensors (+/‒ 0.5% RH) (Lascar, Radiospare, France). Internal atmospheres were stirred using a ventilator (Sunon, Radiospare, France) and all experiments were performed at room temperature.

#### Preparation of the initial cell suspension

The cell concentration of the stationary growth phase culture was checked and adjusted to 1 × 10^8^ CFU/mL through the measurement of the OD_600_. 25 mL of each culture were centrifuged (3,400 g, 10 min at 25°C, Eppendorf 5810 R, Montesson, France) and washed twice with an equal volume of PBS (Phosphate Buffered Saline, Sigma-Aldrich, Saint-Quentin-Fallavier, France). In a final step, the supernatant was removed and cell pellets were suspended in 25 mL of PBS. The final bacterial number was checked by measuring OD_600_. The final cells counts were determined by plating on TSA. Plates were incubated for 24 h at 37°C (+/‒ 0.1°C) and bacterial concentration was expressed in CFU/mL (Colony Forming Unit/mL).

### Rehydration impact after different drying intensities

#### Drying and instantaneous rehydration

**Fig 1:**
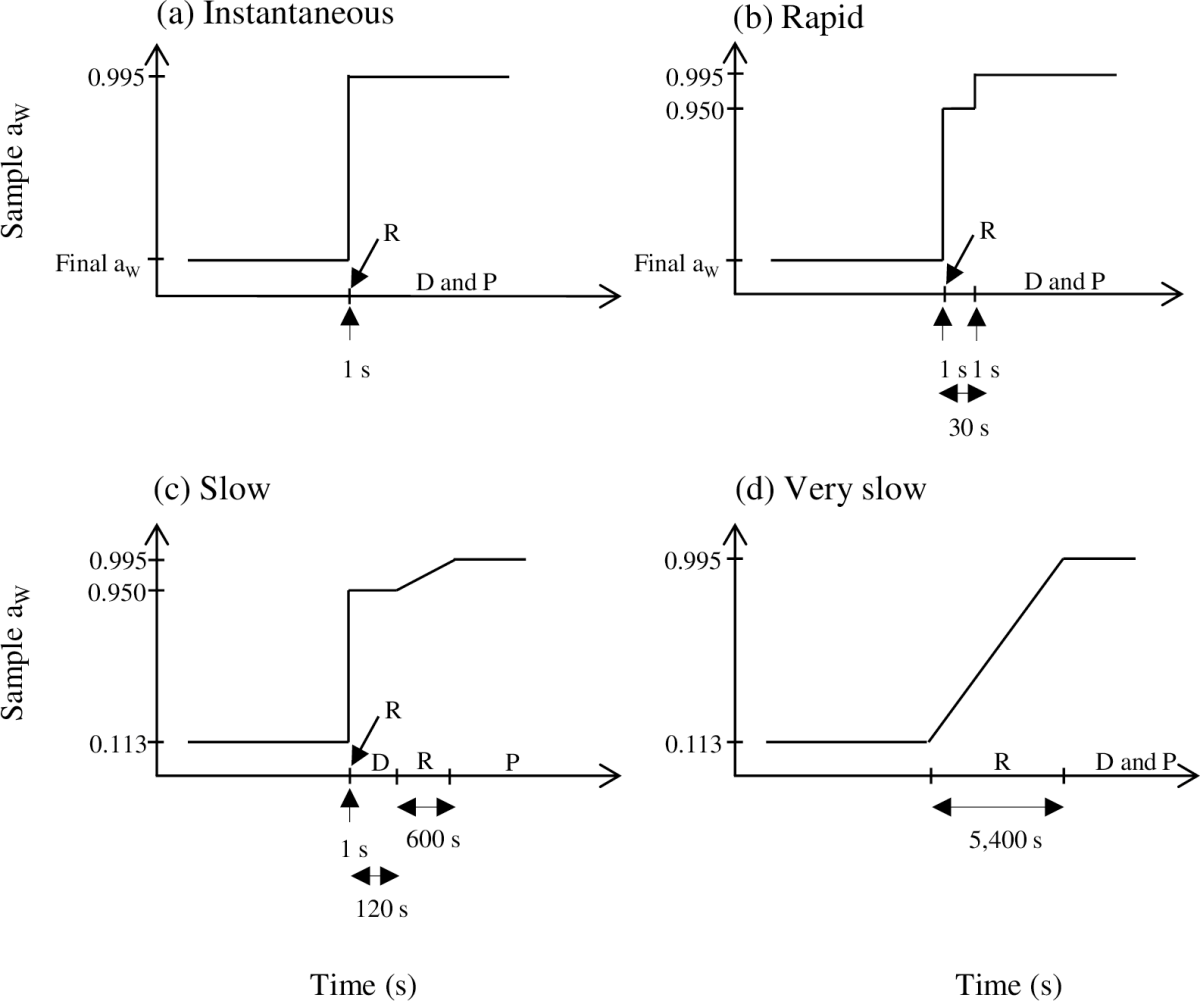
Scheme of different rehydration methods used in this study. (a) Instantaneous rehydration in PBS at 0.995 for 1 s. (b) Rapid rehydration in cPBS (concentrated PBS) at 0.950 (about 1 s), holding time at 0.950 for 30 s and diluting in PBS at 0.995 for 1 s before plating. (c) Slow rehydration in cPBS at 0.950 for 1 s and holding at 0.950 by diluting in cPBS for 2 min before plating during which ramp rehydration (i.e. a progressive evolution of rehydration level) occurred on Petri dishes for about 10 min to reach an a_W_ of 0.995. (d) Very slow rehydration by placing bacteria in a 100% RH atmosphere for 90 min before diluting and plating. R: Rehydration. D: Dilution. P: Plating. Even if only drying at an a_W_ of 0.11 was presented, rehydration presented in (a) and (b) were also performed to reach an a_W_ of 0.58, 0.44 and 0.25 in atmosphere RH at 58%, 44% and 25%.

For each experiment, a droplet (10 μL) of bacterial suspensions was spread in a thin layer onto a glass Petri dish (with 3 cm diameter) which was placed at 58%, 44%, 25% and 11% RH during 90 min. After the desiccation, cells were rehydrated by instantaneous (1 s) addition of 990 μL of PBS (a_W_ = 0.995, checked using an a_W_-meter) and homogenization by pipetting and using a cell scraper (Greiner, Les Ulis, France). Then, cells were diluted in PBS (a_W_ = 0.995) and counted by plating on TSA (a_W_ = 0.995) incubated for 24 h at 37°C (+/‒ 0.1°C) (Fig 1(A)).

#### Drying and rapid rehydration

Droplets (10 μL) of same bacterial suspensions were dried in the same way as for instantaneous rehydration. After the drying, cells were rehydrated by addition of 990 μL of concentrated PBS (noted cPBS, a_W_ = 0.950, checked using an a_W_-meter) and homogenized by pipetting and using a cell scraper (Greiner, Les Ulis, France), corresponding to the first rehydration step. The use of cPBS (a_W_ = 0.950) resulted in the partial rehydration of bacterial cells up to an a_W_ of 0.950 (compared to PBS with a_W_ = 0.995) imposing a supplementary step before total rehydration of the bacterial cells at an a_W_ of 0.995. After 30 s, for the second step, dilution was performed in PBS (a_W_ = 0.995) and counted by plating on TSA (a_W_ = 0.995) incubated for 24 h at 37°C (+/‒ 0.1°C) (Fig 1(B)).

### Drastic drying and slow rehydration rates

Droplets (10 μL) of bacterial suspensions were spread in thin layer on glass. They were placed at 11% RH for 90 min, i.e. the more drastic drying condition. After the drying, cells were rehydrated by two other complementary methods: (i) a slow rehydration was performed such as previously in cPBS but the successive dilution were performed in cPBS to maintain bacteria at 0.950, 10 μL of each dilution was dropped on a TSA plate with an a_W_ = 0.995 (see Fig 1(C) for the corresponding scheme). This drop on the TSA media involved then a linear increase of the cell suspension a_W_ (from 0.950 to 0.995). Cells were counted by plating on TSA incubated for 24 h at 37°C (+/‒ 0.1°C). The second complementary rehydration method is (ii) a very slow rehydration which was performed by placing dried bacteria in an atmosphere maintained at 100% RH (checked using an RH sensor) thanks to vapor transfer from distilled water at room temperature for 90 min permitting bacteria to reach an a_W_ of 0.995. Cells were then homogenized by pipetting and then counted by diluting in PBS (a_W_ = 0.995) and plating on TSA incubated for 24 h at 37°C (+/‒ 0.1°C) (see Fig 1(D) for the corresponding scheme).

### Statistical analysis

The loss of cultivability was expressed in log_10_(N/N_0_), where N represents the bacterial concentration (CFU/mL) after the drying/rehydration cycle and N_0_ represents the bacterial concentration (CFU/mL) before stress, for each condition. All experiments were performed in completely independent triplicates, from independent subcultures and cultures. With the purpose of comparing the various results obtained in this study, first the variance homogeneity (F-test) was tested and then (if p > 0.05) an ANOVA or a *t*-test were performed. For significant ANOVA, Tukey's HSD (Honest Significant Difference) test was achieved. Analyses were performed on R software.

## Results

### Rehydration impact after different drying intensities

**Fig 2:**
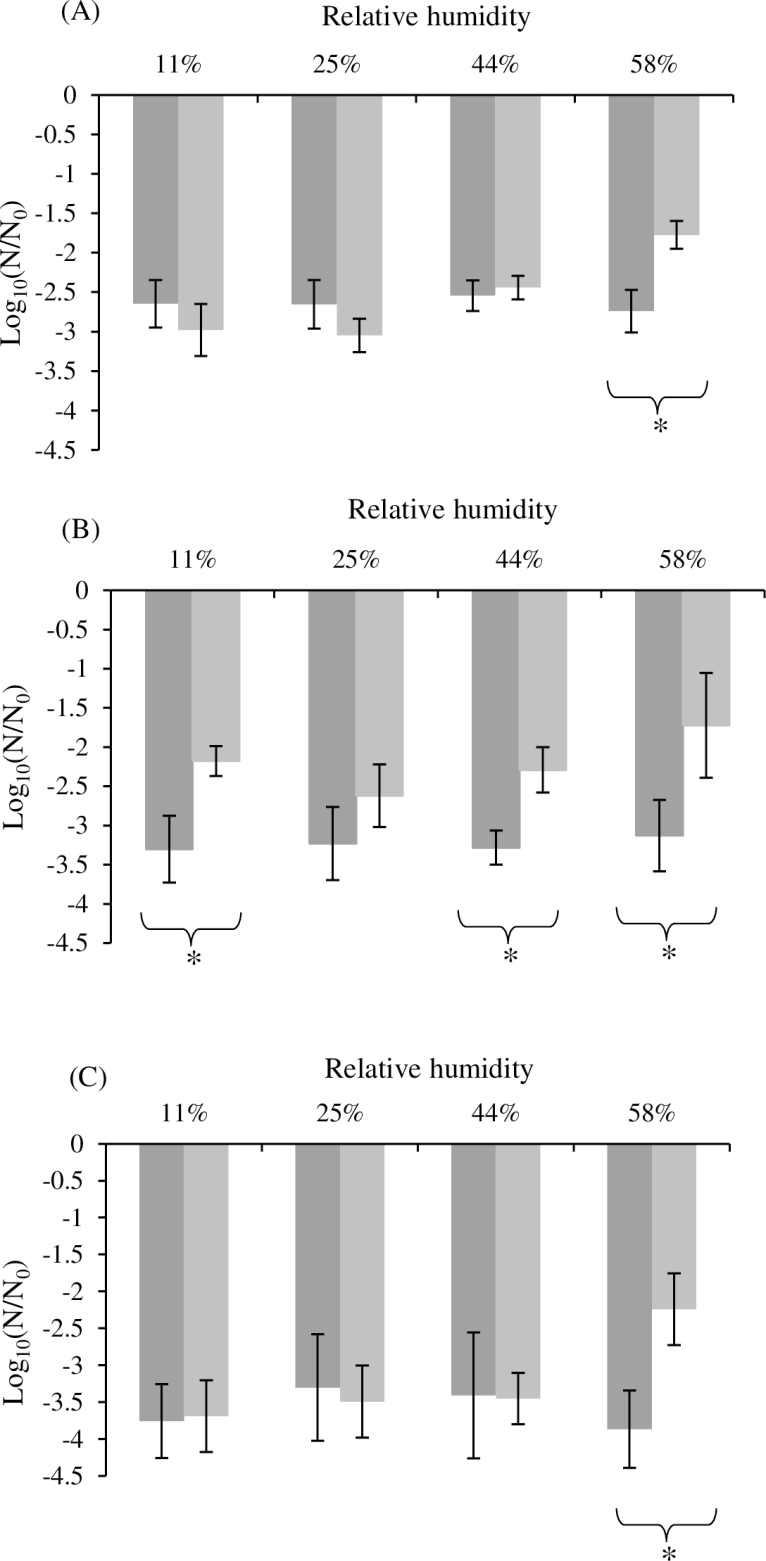
**Impact of rehydration after drying on cultivability for *C*. *sakazakii* (A), S. Typhimurium (B) and *S*. Senftenberg (C).** Dark grey represents the loss of cultivability obtained with instantaneous rehydration and light grey represents the loss of cultivability with rapid rehydration. Error bars represent SD calculated from triplicates and asterisks represent a significant difference between both conditions (*t*-test, p<0.05). The corresponding dataset was presented in S1 Table.

After drying at different RH, bacteria were rehydrated in two different manners: (i) instantaneous rehydration and (ii) rapid rehydration (Fig 1(A) and 1(B)). The loss of cultivability after drying and rehydration is represented in Fig 2. A significant difference was observed in bacteria survival with a significant effect of species (ANOVA, p < 0.05) and a highly significant effect of rehydration (ANOVA, p < 0.01).

Concerning *S*. Typhimurium (Fig 2(B)), the loss of cultivability was higher when instantaneous rehydration was used than when rapid rehydration was used. A significant difference (p < 0.05) was detected between both rehydration conditions after a drying at 11% RH, 44% RH and 58% RH. In the cases of *C*. *sakazakii* (Fig 2(A)) and of *S*. Senftenberg (Fig 2(C)), on the other hand, a significant difference (p < 0.05) was only observed in the 58% RH condition, with a more important loss of cultivability after an instantaneous rehydration than after a rapid rehydration.

### Rehydration impact after a drastic drying

**Fig 3:**
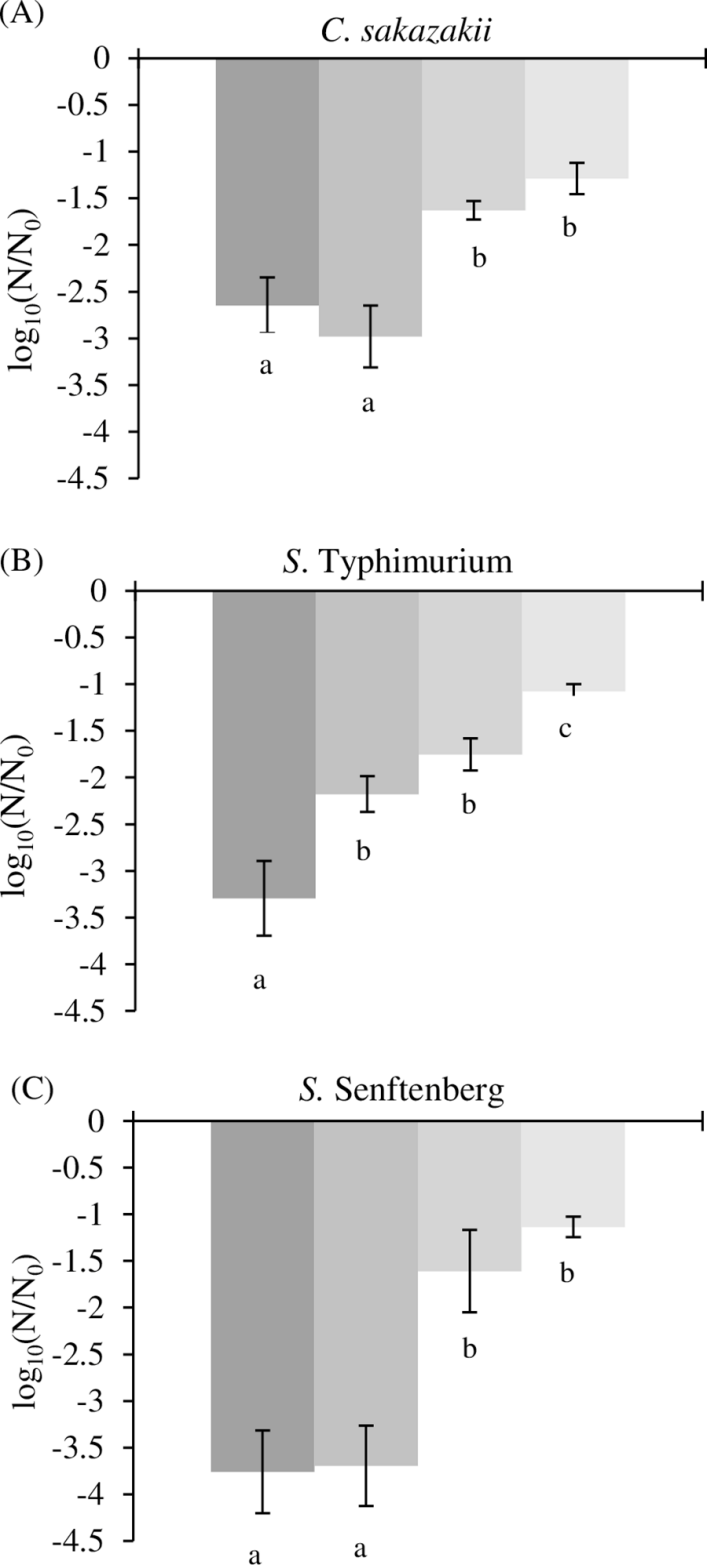
**Comparison of four rehydration kinetics after 90 min of drying in an atmosphere at 11% RH for *C*. *sakazakii* (A), *S*. Typhimurium (B) and *S*. Senftenberg (C).** The loss of cultivability obtained with instantaneous, rapid, slow and very slow rehydration is shown going from darker to lighter. Error bars represent SD calculated from independent triplicates. The letters represent a significant difference (p<0.05) obtained with Tukey's HSD (Honest Significant Difference) test. The corresponding dataset was presented in S2 Table.

In order to explore the effect of rehydration kinetics, two supplementary types of slower rehydration were then performed after drying in drastic conditions (11% RH): (i) slow rehydration and (ii) very slow rehydration (Fig 1(C) and 1D)). The data for loss of cultivability obtained with these new rehydration modes were compared with the previous results obtained with instantaneous and rapid rehydration in the same drying conditions (11% RH) and presented in Fig 3. A highly significant difference was observed for loss of cultivability in the four rehydration kinetics (ANOVA, p < 0.001).

For *S*. Typhimurium (Fig 3(B)), differences among loss of cultivability were observed using instantaneous, rapid, slow and very slow rehydration, with no difference between rapid and slow rehydration. A difference of 2 log was observed when comparing the two extreme rehydration modes: instantaneous and very slow rehydration. Regarding *S*. Senftenberg (Fig 3(C)), differences in loss of cultivability were observed in the different rehydration modes used in this study. Two groups were significantly different (p < 0.05), represented by the two fastest rehydration kinetics and the two slowest rehydration kinetics. The difference between the instantaneous and very slow rehydration modes represented 2.7 log. Concerning *C*. *sakazakii* (Fig 3(A)), results similar to those observed for *S*. Senftenberg were obtained. A difference of 1.25 log was noted between instantaneous and very slow rehydration.

Consequently, the rehydration kinetics was primarily responsible for the loss of cultivability obtained after a drying/rehydration cycle.

## Discussion

These results obtained in our study clearly show that the inactivation usually and totally attributed to drying is in fact due to a combination of drying and of rehydration. This phenomenon has previously been observed in probiotics [15], lactic acid bacteria [16] or yeast [17,18]. However, few studies have considered the rehydration of pathogens, such as in surface rehydration [19] or in model media [20]. In the present case, we could clearly assess the impact of rehydration on dried foodborne pathogens through the observed difference of loss of cultivability between the faster and slower rehydration modes. The slower the rehydration kinetics, the smaller the loss of cultivability was. Such a conclusion could raise two issues. The first one concerns the rehydration performed in classical microbiological food analyses which is often similar to the instantaneous rehydration presented in this study. Indeed, the inactivation of microorganisms during this rehydration would lead to an underestimation of the pathogenic load present in the food and, therefore, to an increased risk for consumers’ health. The second one concerns the different personal consumer practices in terms of dried food product rehydration which could also have important consequences on further pathogen survival in the rehydrated product.

Two phenomena occurring during the drying phase could possibly explain this significant loss of cultivability due to fast rehydration kinetics. One is the cell membrane folding followed by an intracellular vesiculation which occurs during drastic drying when water exits the cell swiftly. Plasma membrane vesiculation has already been observed and reported in previous work, both on yeasts and bacteria [9,18,20]. Indeed, this phenomenon leads to a decrease in the area of membrane. During fast rehydration, water rapidly enters the cell which tends to recover its original volume but the membrane ruptures because of its reduced surface. During the slow rehydration of cells, as used in this work, the internal vesicles would have time to reincorporate the membrane and so to prevent the cell from rupture. The other phenomenon which could be considered to explain our observations is related to osmotic solutes or active ion input in the cell during the first phase of drying [3,21,22]. This solute accumulation would involve a supplementary water input during the rehydration which leads to a greater cell volume than the initial one and so to cell rupture. Indeed, in case of instantaneous rehydration, this water input is not compensated by the simultaneous solute and ion output from the cell which have very slow rates of diffusion across the cell membrane (about 100-fold less than water) [23]. Compared to instantaneous rehydration, the rapid rehydration modes used in this study would give the cell time for these solutes or ions to exit and so to recover its initial volume after total rehydration [9]. It could be interesting to evaluate to what extent each phenomenon destroys the cells.

After drying followed by very slow rehydration, no significant differences were observed between loss of cultivability of *S*. Typhimurium, *S*. Senftenberg and *C*. *sakazakii*, which reached near 1 log for each species (Fig 3). Contrarily, with instantaneous rehydration, a significant difference was observed between the loss of cultivability of each specie, where *C*. *sakazakii* was more resistant than *S*. Typhimurium and *S*. Senftenberg. Until now, *C*. *sakazakii* has been known as an *Enterobacteriaceae* which is highly resistant to drying and more resistant than *Salmonella enterica* [10,22,24]. The results presented in this study suggest that the very slow rehydration mode erases the difference in loss of cultivability of the bacterial species. Consequently, it is possible to think that *C*. *sakazakii* is more resistant to rehydration than *S*. *enterica* and, consequently, not more resistant to drying by itself, maybe explaining by the different capsular properties of the strains.

## Conclusions

Interestingly, the results presented in this study suggest that the impact of drying on the inactivation of pathogen is associated to rehydration kinetics. Indeed, instantaneous rehydration is responsible for the extend inactivation during the drying-rehydration process. Two hypotheses were proposed to understand the role of rehydration kinetics and it seems that appropriate management of rehydration and particularly fast rehydration could enhance the negative effects of drying/rehydration on pathogen cultivability. Rehydration is an important step when cultivability is assessed, impacting the estimation of bacterial contamination and consumer risk. In light of the above conclusions, rehydration after drying has to be normalized during routine microbial analyses in order to avoid underestimating the microbial load in dried food products. During food safety analyses, rehydration procedures need to be standardized. Likewise, instructions for an optimal rehydration during the use of dried food have to be provided to the consumer. In the future, we suggest that rehydration has to be considered with more attention; further works would consider other foodborne pathogens and the influence of the nature of different dried food products.

## Supporting Information

S1 Table Logarithmic reduction of studied pathogens as function of instantaneous and rapid rehydration at four relative humidity levels (11%, 25%, 44% and 58% RH). (XLSX)

S2 Table Logarithmic reduction of studied pathogens as function of four rehydration mods (instantaneous, rapid, slow, and very slow) after drying at 11% RH for 90 min. (XLSX)
